# Epidemiology, Risk Factors, and Outcomes of Respiratory Syncytial Virus Infections in Newborns in Bamako, Mali

**DOI:** 10.1093/cid/ciz157

**Published:** 2019-02-27

**Authors:** Andrea G Buchwald, Boubou Tamboura, Sharon M Tennant, Fadima C Haidara, Flanon Coulibaly, Moussa Doumbia, Fatoumata Diallo, Adama M Keita, Samba O Sow, Karen L Kotloff, Myron M Levine, Milagritos D Tapia

**Affiliations:** 1 Center for Vaccine Development and Global Health, University of Maryland School of Medicine, Baltimore; 2 Centre pour le Developpement des Vaccins, Bamako, Mali

**Keywords:** pneumonia, acute respiratory infection, active surveillance, RSV

## Abstract

**Background:**

Few studies describe the respiratory syncytial virus (RSV) burden in African populations, and most have utilized hospital-based surveillance. In Mali, no community-based studies exist of the incidence or epidemiology of RSV infection. This study provides the first estimates of RSV incidence in Mali.

**Methods:**

In a cohort of infants enrolled in a clinical trial of maternal influenza vaccination, we estimate incidence of RSV-associated febrile illness in the first 6 months of life and identify risk factors for RSV infection and progression to severe disease. Infants (N = 1871) were followed from birth to 6 months of age and visited weekly to detect pneumonia and influenza-like illness. Baseline covariates were explored as risk factors for RSV febrile illness and RSV pneumonia or hospitalization.

**Results:**

Incidence of RSV illness was estimated at 536.8 per 1000 person-years, and 86% (131/153) of RSV illness episodes were positive for RSV-B. RSV illness was most frequent in the fifth month of life and associated with having older mothers and with lower parity. The incidence of RSV-associated hospitalizations was 45.6 per 1000 person-years. Among infants with RSV illness, males were more likely to be hospitalized. The incidence of RSV pneumonia was 29 cases per 1000 person-years.

**Conclusions:**

In the first 6 months of life, Malian infants have a high incidence of RSV illness, primarily caused by RSV-B. Prevention of early RSV will require passive protection via maternal immunization in pregnancy. Mali is the first country where RSV-B has been identified as the dominant subtype, with potential implications for vaccine development.

## Teaser

This first report of community-based surveillance for respiratory syncytial virus (RSV) infection in Mali showed that RSV-B was the dominant subtype, in contrast to other regions. Incidence was high early in life, before infant vaccination is a viable strategy.

Yearly, approximately 34 million respiratory syncytial virus (RSV)-associated acute respiratory infections (ARIs) worldwide in children <5 years of age lead to 3 million hospitalizations for severe RSV [1, 2]. RSV accounts for 22% of all ARIs worldwide, and the majority (96%) of RSV illness occurs in low-resource countries [1]. Incidence of RSV-associated illness is highest among infants <5 months of age, when presence of maternally derived antibodies prevents effective vaccination. However, incidence and mortality likely vary substantially from year to year and between settings.

Despite the putative high burden of ARIs in Africa, there are few publications studying ARIs in African populations. Multiple studies have used passive hospital-based surveillance to estimate incidence of ARI in Africa [3, 4], finding that ARIs cause approximately 8% of all deaths among South African children <5 years of age [3]. However, few studies have used active community-based surveillance [5–7]. Hospital-based surveillance greatly underestimates the incidence and risk of RSV-associated ARI; the rate of RSV-associated severe disease may be 4–5 times higher in the community than the rate of RSV hospitalization [6]. Increased understanding of the epidemiology of RSV disease is necessary for designing vaccination strategies in the wake of soon-to-be-available RSV vaccines for preventing early infant morbidity [8].

In Mali, ARI accounts for 13% of hospital-recorded morbidity [4] and RSV contributes substantially to this disease burden, with 25% of hospitalized pneumonia cases among children under five and up to 44% of hospitalizations for acute lower respiratory infections positive for RSV [2, 9]. Despite high rates of RSV in neonates, most published data are for children <5 years of age. There is an urgent need for precise data on younger ages, particularly infants in the first 6 months of life, and from community-detected cases [7]. Here we report on the epidemiology, incidence, risk factors, and outcomes of RSV-associated ARI from community-based active surveillance in Bamako, Mali.

## METHODS

### Study Design

This study of RSV epidemiology utilizes a cohort from a clinical trial on the efficacy of maternal influenza vaccination for prevention of influenza in infants [10]. We conducted a randomized trial, whereby pregnant Malian women were allocated to receive either trivalent influenza vaccine or quadrivalent meningococcal conjugate vaccine. Enrolled pregnant women and their newborns were followed until infants reached 6 months of age.

From September 2011 to April 2013, 4193 pregnant women were enrolled and followed with weekly home visits to assess newborn infants for pneumonia and influenza-like illness (ILI) [10]. Pneumonia was classified following World Health Organization criteria [11]. Among infants without pneumonia, the case definition of ILI was met if a fever was reported or observed by a clinician and either (1) fever presented without a source or (2) fever presented in combination with ARI (febrile ARI). ARI was defined as presence of any of runny nose, nasal congestion, cough, difficulty breathing, pus from ears, or wheezing. Details of sampling are shown in the Supplementary Materials.

Infants born from October 2012 to May 2013 were selected for the current analysis based on known RSV circulation in the area (A. Driscoll, personal communication, 2017). Samples from these infants were selected to be tested for RSV as follows: all samples from infants with pneumonia or fever without a source, and 30% of specimens from infants with febrile ARI who were negative for influenza. Among infants born October 2012–May 2013 who experienced any ILI or pneumonia, 37% of infants had samples tested for RSV and other pathogens. The Center for Vaccine Development-Mali Molecular Diagnostics Unit utilized the Fast Track Diagnostics respiratory pathogens 33 reverse-transcription polymerase chain reaction (RT-PCR) kit (FTD33; Junglinster, Luxembourg), and RSV-positive samples were further tested with the RealStar RSV RT-PCR kit (Altona Diagnostics) to differentiate between subtypes A and B.

### Predictors of RSV Infections

To examine predictors of RSV illness, a nested case-control study design was used. Cases were infants who experienced pneumonia or febrile ARI and had samples from the episode subsequently test positive for RSV. Cases were matched to 2 sets of controls who were under active surveillance at the time the RSV case occurred: (1) infants with any RSV-negative ILI or pneumonia within 2 weeks of the RSV case (sick controls), and (2) infants without any ILI or pneumonia during RSV season (healthy controls). Sick controls were defined as infants who (1) experienced pneumonia or ILI with an onset of fever within 2 weeks of onset of fever in the case, (2) received an RSV test for that pneumonia or ILI, (3) never tested positive for RSV, and (4) had no previous ILI that went untested for RSV. Up to 2 sick controls were matched to each case. For each RSV case, sick controls were matched first and then healthy controls were matched to cases by birth date as a simple random sample of eligible controls, in chronological order. A given control could not be matched to >1 case. The matches were made in chronological order, according to the date of RSV case fever onset and RSV case age.

### Statistical Analysis

#### RSV Incidence

Baseline covariates were compared among infants with pneumonia or ILI between those included and those excluded (untested for RSV) to check for bias in the selection of infants with pneumonia or ILI into the study population. Categorical covariates were tested using χ^2^ tests, and continuous covariates were tested using *t* tests with Satterthwaite variances.

Incidence of first RSV illness was calculated. Infants included in the denominator for RSV incidence were those with ILI or pneumonia tested for RSV and a simple random sample of infants born from October 2012 to May 2013 without ILI during follow-up. Infants without ILI were selected with 0.37 probability of being included in the denominator, to match the proportion of infants with ILI or pneumonia included in the population. Follow-up time for all infants began at birth. For infants with RSV-positive illness, follow-up time ended at the time of RSV-positive ILI or pneumonia. For infants with RSV-negative illness, follow-up time ended either at the first untested ILI or, if all samples were tested, at the final study visit. Follow-up time for infants without ILI or pneumonia ended at the final study visit.

### Predictors of RSV Infections and Outcomes

The following covariates were explored as potential predictors of RSV infection and predictors of RSV outcomes: Maternal age, parity, maternal education level, household crowding, socioeconomic status, maternal vaccination group, infant sex, infant gestational age at birth, and infant birth weight. Maternal socioeconomic status was calculated using principal components analysis with household wealth indicators [12], and then categorized into low (bottom 25%), medium (middle 50%), and high (top 25%). Maternal education level was dichotomized by completion of primary school. Household crowding was defined as the number of people in the household divided by the number of rooms and was dichotomized as <3 people per room vs ≥3 people per room. Gestational age at birth was calculated using the best available measure in the data, with the order of priority being (1) first trimester ultrasound, (2) late ultrasound, (3) report of last menstrual period, (4) New Ballard Score [13] at birth, and (5) uterine height at enrollment. Gestational ages estimated at >42 weeks were coded as term. For examining association with pneumonia incidence, gestational age was dichotomized as either preterm (born before 37 weeks of gestational age) or term. Birthweight was dichotomized as either low birth weight (<2500 g) or not.

For predictors of RSV infections, McNemar χ^2^ test was used to compare categorical variables between control groups and RSV cases and Wilcoxon signed-rank tests were used to compare the distribution of continuous predictor variables between control groups and RSV cases. To explore the association between covariates and RSV pneumonia or hospitalization among infants with RSV infection, χ^2^ tests or Fisher exact tests were used. All statistical analysis was done using SAS version 9.4 software.

## RESULTS

**Figure 1.**
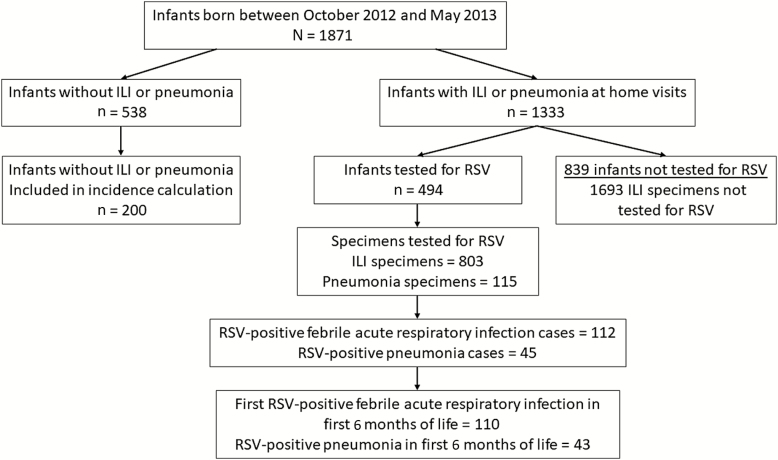
Flowchart showing selection of specimens for testing for respiratory syncytial virus infection and outcomes. Abbreviations: ILI, influenza-like illness; RSV, respiratory syncytial virus.

There were 1871 infants born between October 2012 and May 2013 and eligible for inclusion in this analysis (Figure 1). Among those, 1333 of 1871 (71%) had ILI or pneumonia during the first 6 months of life and 494 of 1333 (37%) infants with ILI or pneumonia had samples tested for RSV. Of 1871 infants, 538 (29%) experienced no ILI or pneumonia during follow-up. Among infants with no ILI or pneumonia during follow-up, 200 of 538 (37%) infants were randomly sampled to be included in the denominator for follow-up time.

Infants with all ILI that who were not tested for RSV (n = 839) were excluded from the incidence calculation. There were no statistically significant differences between infants included or excluded from the incidence calculation (Table 1).

**Table 1.** Population Characteristics of Infants With Pneumonia or Influenza-like Illness by Testing Status

Characteristic	Infants/Specimens Tested for RSV	Infants/Specimens Not Tested for RSV
Maternal characteristics, No.	494	839
Age, y, mean (SD)	25.0 (5.99)	24.8 (5.94)
Less than primary education	416 (84.2%)	686 (81.8%)
Primary or more education	78 (15.8%)	153 (18.24%)
Parity, mean (SD)	3.2 (2.05)	3.3 (2.12)
Wealth index, mean (SD)^a^	0.12 (3.35)	0.06 (3.50)
Influenza vaccine arm	248 (50.2%)	414 (49.3%)
Infant characteristics, No.	494	839
Male sex	253 (51.2%)	424 (50.5%)
Gestational age at birth, wk, mean (SD)	39.5 (2.37)	39.5 (2.12)
Birth weight, g, mean (SD)	3055 (459)	3069 (418)
Receipt of standard vaccines^b^ at 6 wk, %	90.3%	93.1%
Illness characteristics, No.	918	1693
Fever without a source	19 (2.1%)	12 (0.7%)
Fever + acute respiratory infection	784 (85.4%)	1681 (99.3%)
Pneumonia	115 (12.5%)	0 (0.0%)

Data are presented as No. (%) unless otherwise indicated. Influenza-like illness is defined as either presence of fever without a source or presence of fever plus symptoms of acute respiratory infection.

Abbreviations: RSV, respiratory syncytial virus; SD, standard deviation.

^a^Wealth index calculated using principal components analysis to have a population mean of 0.0 (SD, 3.6).

^b^In Mali, infants receive diphtheria-pertussis-tetanus/*Haemophilus influenzae* type b/hepatitis vaccine, oral polio vaccine, and pneumococcal conjugate vaccine at 6 weeks.

### RSV Incidence

Among 1871 included infants, there were 2611 ILI or pneumonia samples, 918 of 2611 (35%) were tested for RSV. Of tested samples, 157 of 918 (17.1%) were positive for RSV. All 115 pneumonia episodes occurring between October 2012 and May 2013 were tested, and 45 (28.7%) were positive for RSV. One infant had 2 RSV episodes in the first 6 months of life, but only the first RSV episode was included for incidence estimates, and 3 episodes of RSV (2 of which presented as pneumonia) occurred after 6 months of age, leaving 153 first RSV illness episodes in the first 6 months of life included in incidence estimates.

**Figure 2.**
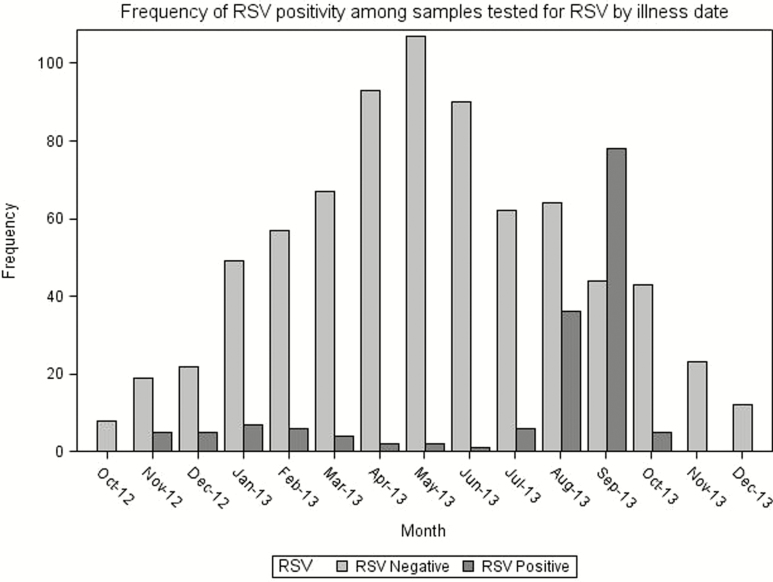
Epidemiologic curve showing frequency of respiratory syncytial virus–positive samples over the course of the study. Abbreviation: RSV, respiratory syncytial virus.

**Figure 3.**
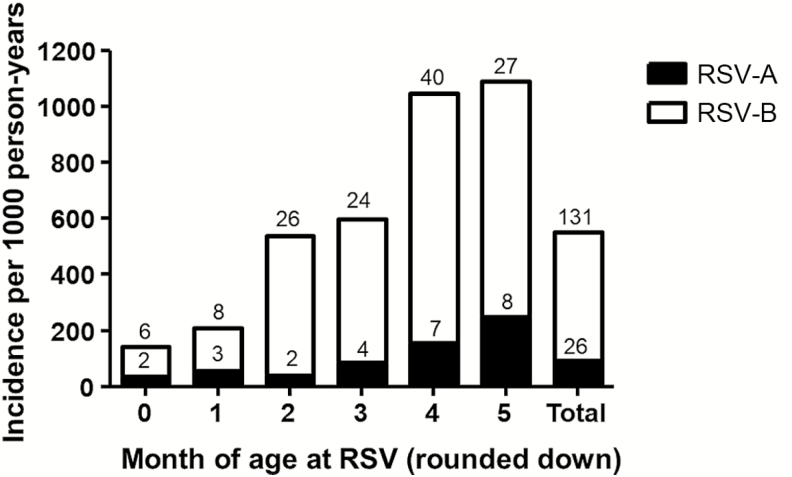
Incidence of strain-specific respiratory syncytial virus–associated illness by month of age. Bar height indicates incidence rate per 1000 person-years. The number inside the bar indicates the number of cases contributing to the incidence rate. Abbreviation: RSV, respiratory syncytial virus.

Overall incidence of RSV illness was 536.8 per 1000 child-years of follow-up time. The majority of RSV illness was positive for RSV-B (131/153), and the incidence of RSV-B was 459.6 per 1000 child-years. The plurality of RSV cases occurred in August and September (Figure 2) and incidence was highest in the fifth month of life (Figure 3).

### Clinical Presentation and Outcomes of RSV

The mean age at presentation for RSV illness was 116 days (range, 12–183 days). Of 153 first cases of RSV illness in the first 6 months of life, 45 (29.4%) presented with pneumonia and 108 (70.6%) presented with febrile ARI. Among RSV pneumonia cases, 13 (8.5%) were hospitalized, and 1 (0.7%) died. The incidence rate of RSV pneumonia was 29 cases per 1000 person-years of follow-up time. RSV-B was detected in 35 (78%) RSV pneumonia cases and there was no statistical difference in the proportion of pneumonias by RSV subtype (*P* = .14) (Table 2). The incidence rate of RSV hospitalizations was 45.6 per 1000 years of follow-up time. The single RSV pneumonia–associated death occurred at an age of 68 days. Among infants with RSV, there was no association between covariates and pneumonia; male infants were more likely to be hospitalized (Table 3).

**Table 2.** Incidence of Respiratory Syncytial Virus (RSV) Pneumonia by Age and by Strain of RSV

Month of Age at Pneumonia (Rounded Down)	RSV Pneumonia, No.	RSV-B Pneumonia, No.	Follow-up Time, y	RSV Pneumonia Incidence^a^	RSV-B Pneumonia Incidence^a^
0	2	2	263	7.61	7.61
1	5	4	261	19.15	15.32
2	10	8	259	38.62	30.89
3	4	3	256	15.60	11.70
4	12	10	254	47.31	39.42
5	10	7	186	53.78	37.64
Total	43	34	1479	29.08	22.99

Abbreviation: RSV, respiratory syncytial virus.

^a^Incidence per 1000 person-years.

**Table 3.** Predictors of Pneumonia, Hospitalization, and Death Among Infants With Respiratory Syncytial Virus Infections

Variable	Pneumonia No. (%)	*P* Value	Hospitalizations No. (%)	*P* Value
Influenza vaccine	26 (33)	.21	9 (11)	.25
Meningococcal vaccine	17 (23)		4 (5)	
Maternal SES				
Low SES	10 (28)	.61	3 (8)	.54
Med SES	24 (31)		5 (6)	
High SES	9 (23)		5 (12)	
Maternal education level				
No primary	36 (29)	.89	12 (10)	.47
Primary or more	7 (26)		1 (4)	
Maternal age ≤24 y	18 (29)	.84	5 (8)	.77
Maternal age >24 y	18 (26)		7 (10)	
<3 people/room	33 (33)	.09	10 (9)	.76
≥3 people/room	6 (17)		3 (7)	
Low birth weight (<2500 g)	3 (38)	.54	2 (25)	.14
Birth weight ≥2500 g	40 (28)		11 (8)	
Preterm (gestational age ≤37 wk)	5 (50)	.2	1 (10)	.93
Gestational age >37 wk	38 (30)		11 (9)	
Male sex	24 (32)	.37	10 (13)	.04
Female sex	19 (24)		3 (4)	
Total	43		13	

*P* value for association between covariates and risk of outcome using Fisher exact test for 2 × 2 comparisons and χ^2^ test for association between maternal SES and risk of outcome. Maternal age was missing for 21 infants.

Abbreviation: SES, socioeconomic status.

### Predictors of RSV

Mothers of healthy controls had greater parity and were younger than mothers of RSV cases (*P* = .03, and *P* = .02, respectively). Sick controls came from families with significantly lower wealth than RSV cases (*P* = .04) (Table 4).

**Table 4.** Predictors of Respiratory Syncytial Virus Illness by Case/Control Status

Characteristic	RSV Cases (n = 146)	Sick Controls (n = 190)	*P* Value	Healthy Controls (n = 260)	*P* Value
Maternal characteristics					
Influenza vaccine arm, No. (%)	82 (56)	101 (53)	.66	131 (50)	.30
Parity, median (IQR)	3 (1–4)	3 (1–4)	.07	3 (2–5)	.03
Wealth index^a^, mean (SD)	0.65 (3.5)	–0.09 (3.5)	.04	0.52 (3.9)	.47
Less than primary education, No. (%)	120 (82)	156 (82)	.93	203 (78)	.37
Primary or more education, No. (%)	26 (18)	34 (18)		57 (22)	
Household crowding, people/room, mean (SD)	2.8 (2.1)	2.8 (1.7)	.46	2.8 (1.5)	.41
Age, y, mean (SD)	25.6 (6.2)	24.9 (5.9)	.36	24.0 (5.9)	.02
Infant characteristics					
Male sex, No. (%)	71 (49)	101 (53)	.44	128 (49)	.92
Gestational age, wk, mean (SD)	39.9 (2.2)	39.4 (2.3)	.06	39.7 (2.3)	.31
Birth weight, g, mean (SD)	3124 (476)	3063 (453)	.40	3070 (431)	.70

*P* value for comparing controls to cases using Wilcoxon test for continuous variables and χ^2^ test for categorical variables.

Abbreviations: IQR, interquartile range; RSV, respiratory syncytial virus; SD, standard deviation.

^a^Wealth index calculated using principal components analysis to have a population mean = 0.0 (SD, 3.6).

### Coinfections

All RSV-positive samples yielded at least 1 other positive result on FTD33 kit testing. Among 157 RSV illnesses, other commonly detected pathogens included *Streptococcus pneumoniae* (78%), *Moraxella catarrhalis* (67%), *Klebsiella pneumoniae* (66%), *Haemophilus influenzae* (51%), *Pneumocystis jirovecii* (28%), *Staphylococcus aureus* (26%), rhinovirus (18%), *Legionella* (15%), parainfluenza (13%), and bocavirus (13%). Among 45 RSV pneumonias, other detected pathogens included *M. catarrhalis* (78%), *S. pneumoniae* (82%), *K. pneumoniae* (58%), *H. influenzae* (53%), *S. aureus* (24%), and rhinovirus (16%). Twenty-one infants in the study cohort (including 5 who were RSV positive) had blood cultures taken at the time of the illness; all had a negative result. Of all ILI episodes in the study, 72% received antibiotics, this proportion did not differ by illness severity or RSV status.

## Discussion

Incidence of community-detected RSV illness was high in a cohort of Malian infants, and the RSV-B subtype comprised the majority of RSV infections. We found that low parity and older maternal age were risk factors for RSV illness among infants in the first 6 months of life. These are the first reported data on community-detected RSV illness in Mali and are important for our understanding of RSV epidemiology and burden in the region.

The high rate of early-life RSV infections, before infants can be successfully immunized against infection, has broad consequences for RSV vaccine design and implementation. RSV infections occurred starting in the first month of life and nearly half of all cases were within the first 4 months of life. Early-life RSV cases may be prevented by boosting passive immunity, either through maternal immunization strategies or through administration of a birth dose of an extended half-life monoclonal antibody, strategies that are currently under study [7]. Research on maternal immunization has found that maternal antibody–derived protection against infection can last to the fourth month of life, after which conventional immunization strategies can be utilized [10].

Using active surveillance with weekly in-home visits, this study detected mild cases of RSV illness that would not have necessarily prompted visits to health clinics or hospitals. Nevertheless, mild illnesses contribute to RSV transmission and are relevant for vaccine efficacy. Underestimating the incidence of mild illness leads to underestimating the burden of disease and the basic reproduction rate. Both of these values have consequences for funding and public health decisions, such as the necessary proportion of individuals to vaccinate in a population [14]. The epidemiology of mild RSV illness may differ substantially from previously described epidemiology of severe RSV illness. Previous incidence estimates of RSV illness in Mali come from hospital-detected RSV cases alone, where incidence was estimated at 58 cases per 1000 child-years [2]. Incidence of RSV-associated hospitalizations was similar in our cohort (46 cases per 1000 child-years), but this represented only 9% of total RSV illnesses detected. Despite a rate of RSV hospitalizations, comparable to previous studies, the mortality rate from RSV hospitalizations was relatively low compared to previous findings of ≥12% [3]. This is likely related to the study design: This cohort was followed as part of a clinical trial, thus the infants were all relatively healthy, closely followed, and well cared for.

Two previous studies in Africa have conducted home-based active surveillance for RSV illness; in Nigeria, incidence of RSV ARI was 116 per 1000 child-years [5], and in Kenya, incidence was 104 per 1000 child-years [6]. In Mali, we detected approximately 5 times as many RSV infections than either previous study; numerous differences in study design may have led us to a higher estimate of RSV disease incidence. Incidence in both previous studies was reported for all children <1 year of age, possibly diluting the estimate as incidence may be higher before 6 months. Both previous studies used immune-based assays to detect RSV. PCR-based assays are inherently more sensitive, and the use of a PCR-based assay may have caused us to detect RSV in sick infants even when the illness was not directly caused by RSV infection. A high rate of coinfections was found; thus, many illnesses we assigned as RSV disease may have been due to other pathogens, leading to an overestimate of RSV disease incidence. Our findings are similar to previous data from Mali, where, with similar proportion of coinfections, only *S. pneumoniae*, human metapneumovirus, and RSV were statistically associated with disease [9]. We tested infants born during a known period of RSV circulation and oversampled severe cases of disease in our population when choosing infants to test for RSV, both potentially leading to an overestimate of RSV incidence in our population. Alternately, the current case definition for RSV ARI does not require presence of fever [15]; by selecting only for febrile illnesses and not utilizing serology to detect additional RSV cases, we may have underestimated RSV incidence [16].

Previous studies from around the world have identified numerous risk factors for RSV and severe RSV disease, including preterm birth, low birth weight, male sex, and household crowding [3, 17–19]. In contrast, in Mali we found that maternal characteristics were more important in predicting mild RSV infection; cases were more likely to be born to older mothers with lower parity, possibly as a result of decreased recent maternal exposure to RSV. Birth weight and gestational age did not vary between RSV cases and healthy controls in our study population. However, there were few severe outcomes of RSV infections, potentially preventing detection of a difference between groups. Looking among infants with RSV infection, male sex predicted severe disease presentation, in agreement with previous literature. Additionally, RSV-positive infants with low birth weight and born preterm had increased frequency of both pneumonia and hospitalizations; however, due to low outcome numbers, this difference was not significant.

RSV-A subtype infections are thought to be more frequent than RSV-B [20]. We found that 86% of all RSV cases in our population were positive for RSV-B and subtype was not associated with severity of illness. Studies on RSV subtypes from around the world have consistently found the RSV-A subtype to be more common; additionally, RSV-A is thought to have higher transmissibility than RSV-B [21–23]. While previous studies have found much variability in subgroups, and others have described a predominance of RSV-B during outbreaks [24], this is the first burden of disease study finding RSV-B as the dominant subtype, suggesting that RSV subtype selection may vary more between populations than previously thought.

Lack of funding prevented us from testing all infant ARI samples for RSV, and samples from pneumonia cases and hospitalizations were oversampled for testing. Thus, the proportion of RSV cases leading to pneumonia or hospitalization in the cohort is overestimated and should not be generalized. Despite potential overestimation of the contribution of RSV to illness, this study benefits from several strengths. All RSV infections met a stringent case definition, in contrast to previous studies that defined the outcome as ARI alone. Intensive active surveillance with weekly home visits and a highly sensitive detection method increased our ability to detect RSV infections in the population. This is the first report of RSV burden in Mali and represents an important contribution to the understanding of RSV infection in the region.

We found a high incidence of RSV infection early in life among Malian newborns. RSV-B was the dominant subtype of RSV infections in Mali, suggesting that characterization of the molecular epidemiology of RSV in additional high-incidence countries may be necessary for successful vaccine design. These data represent the first community-detected estimates of RSV illness in Mali and provide valuable information for the future design of RSV vaccination strategies.

## Supplementary Data

Supplementary materials are available at *Clinical Infectious Diseases* online. Consisting of data provided by the authors to benefit the reader, the posted materials are not copyedited and are the sole responsibility of the authors, so questions or comments should be addressed to the corresponding author.

ciz157_suppl_Supplementary_Material

## Notes

***Acknowledgments.***The authors thank the clinical study team, participants’ families, Dr William Blackwelder, Dr Nicholas Schluterman, and Deanna Toema.

***Financial support.***This work was supported by the Bill & Melinda Gates Foundation (grant number OPP1002744).

***Potential conflicts of interest.***The authors: No reported conflicts of interest. All authors have submitted the ICMJE Form for Disclosure of Potential Conflicts of Interest. Conflicts that the editors consider relevant to the content of the manuscript have been disclosed.
