# Supplementary material for: Epidemiology, Risk Factors, and Outcomes of Respiratory Syncytial Virus Infections in Newborns in Bamako, Mali
Source: Clin Infect Dis. 2019 Feb 27;70(1):59–66. doi: 10.1093/cid/ciz157 (PMC6912158; doi:10.1093/cid/ciz157)
Supplement: ciz157_suppl_Supplementary_Material [file ciz157_suppl_supplementary_material.docx]

Epidemiology, Risk Factors, and Outcomes of Respiratory Syncytial Virus Infections in Newborns in Bamako, Mali

Supplemental Material:

**Detailed methods for collection and processing of nasopharyngeal swabs:**

Swabs used for nasal samples were Copan Minitip Flocked Dry Swabs. They were put into Universal Transport media from Becton Dickinson. To take sample, the infant’s head was tilted back and the swab was inserted into nare until there was resistance. The swab was then rotated 3 times – e.g. once to the right, then the left and then the right again. The procedure was then repeated if necessary in the other nare and the swab was placed in the viral transport media. The pharyngeal sample was collected with the aid of a tongue depressor and the tonsils were swabbed. The samples were labelled and then stored at 2 – 8˚ C while the temperature was monitored. All personnel were trained at the start of the study and supervised.
